# Design Considerations of an ITO-Coated U-Shaped Fiber Optic LMR Biosensor for the Detection of Antibiotic Ciprofloxacin

**DOI:** 10.3390/bios13030362

**Published:** 2023-03-09

**Authors:** Vikas, Paola Saccomandi

**Affiliations:** Department of Mechanical Engineering, Politecnico di Milano, 20156 Milan, Italy; vikas@polimi.it

**Keywords:** U-shaped fiber, lossy mode resonance, antibiotics, ciprofloxacin, sensitivity, figure of merit

## Abstract

The extensive use of antibiotics has become a serious concern due to certain deficiencies in wastewater facilities, their resistance to removal, and their toxic effects on the natural environment. Therefore, substantial attention has been given to the detection of antibiotics because of their potential detriment to the ecosystem and human health. In the present study, a novel design of indium tin oxide (ITO) coated U-shaped fiber optic lossy mode resonance (LMR) biosensor is presented for the sensitive detection of the antibiotic ciprofloxacin (CIP). The performance of the designed U-shaped LMR sensor is characterized in terms of its sensitivity, full width at half maximum (FWHM), the figure of merit (FOM), and the limit of detection (LOD). For the proposed U-shaped LMR sensing probe, the various crucial factors such as the thickness (d) of the ITO layer, sensing region length (L), and bending radius (R) are optimized. The thickness of the ITO layer is optimized in such a way that two LMR curves are observed in the transmission spectrum and, thereafter, the performance parameters are evaluated for each LMR. It is observed that the designed U-shaped LMR sensor with optimized parameters shows an approximately seven-fold enhancement in sensitivity compared to the straight-core fiber optic LMR sensor. The numerical results revealed that the designed U-shaped fiber optic LMR biosensor can provide a maximum sensitivity of 17,209.9 nm/RIU with the highest FOM of 91.42 RIU^−1^, and LOD of 6.3 × 10^−5^ RIU for the detection of CIP hydrochloride in the concentration range of 0.001 to 0.029 mol∙dm^−3^. Thus, it is believed that the designed LMR biosensor can practically explore its potential use in environmental monitoring and biomedical applications and hence, opens a new window of opportunity for the researchers working in the field of U-shaped fiber optic LMR biosensing.

## 1. Introduction

**Figure 1:**
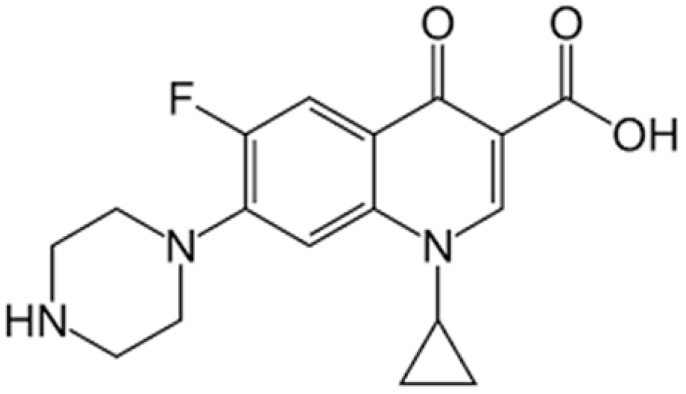
Chemical structure of ciprofloxacin (C_7_H_18_FN_3_O_3_).

Antibiotics are among the key discoveries of the last century that largely transformed the treatment of infections in a significant way. These are the chemical substances that are synthesized to eradicate or inhibit the growth of microorganisms and their derivatives in humans and animals, such as bacteria, fungi, viruses, and protozoa [1]. However, once they leave our bodies and are circulated into waterways, they can harm the environment. This is because some antibiotics given to humans and animals are excreted unchanged in feces and urine. In the case of waste from animals, manure is abundant in nutrients and is mostly used as fertilizer on crop fields, leading to direct contamination of the environment with both antibiotic residues and resistant bacteria. Therefore, due to their good water solubility and stubbornness in the natural environment over a long period of time, antibiotics can affect endocrine activity, metabolism, and development in humans [2]. Worldwide, the annual consumption of antibiotics exceeds 100,000 tons [3], but there are still no regulations regarding the levels of antibiotics in surface water. In June 2018, the European Commission added the antibiotics amoxicillin and ciprofloxacin (CIP) to the updated 3rd Watch List under the Water Framework Directive [4]. Among them, CIP is the most significant antibiotic, which belongs to the family of drugs called quinolones, and its chemical structure is presented in Figure 1. CIP is mostly used to treat the infections of sinuses, bones, prostate, lungs, and bladder, typhoid fever, and sexually transmitted diseases [5].

The overuse of these antibiotics has raised the concentrations of antibiotic residues in both groundwater and surface water [6,7,8]. Citing these purposes, the detection of antibiotic contaminants in water has drawn considerable interest from researchers in the past few years [9,10]. There have been numerous instrumental methods and techniques developed so far for the detection of antibiotics, such as radioimmunoassay [11], capillary electrophoresis [12], liquid chromatography [13], ion mobility spectroscopy [14], mass spectrometry [15], Raman spectroscopy [16], and many more [17,18]. However, these techniques generally need skilled operators and complex laboratory equipment, which are time-consuming and a bit expensive. Thus, there seems to be an imperative need to develop convenient and cost-effective techniques for the detection of antibiotics in water. Related to this, fiber optic sensors (FOSs) offer various important features including high sensitivity, better accuracy, immunity to electromagnetic interference, cost-effectiveness, fast response, low weight, easy processing, compatibility, multiplexing, flexibility, and their ability to operate in a harsh environment [19]. As a result, FOSs have been the subject of intense research for many years and have found ubiquitous applications in physical, chemical, medical, and environmental sensing applications [20,21,22,23,24,25,26,27,28]. Different kinds of FOSs have also been utilized for the detection of several antibiotics including ciprofloxacin, vancomycin, beta-lactam, benzylpenicillin, sulphonamides, etc. [29,30,31,32]. In 2014, Korposh et al. fabricated an optical fiber long-period grating (LPG) sensor functionalized with molecularly imprinted polymer nanoparticles (NPs) for the specific detection of vancomycin [29]. The principle of the designed sensor was based on the interaction of NPs with the antibiotics, resulting in a change in the refractive index (RI) at the sensing interface. Recently, an optical fiber-based nanoplasmonics biosensor was proposed for the sensitive detection of ampicillin combined with gold NPs, and the limit of detection (LOD) was achieved at 0.74 ppb [30]. Later, Huang et al. designed a novel fluorescent FOS for the detection of CIP in water samples utilizing molecularly imprinted NPs composite hydrogel detector [31]. The obtained results showed a linear relationship of the fluorescent intensity with the concentration of CIP having a correlation coefficient of 0.9959 and LOD of 6.86 µM. After this, Wei et al. presented a highly sensitive evanescent wave optical fiber aptasensor for the online, continuous, and selective detection of sulphonamides in environmental water [32].

Recently, FOSs based on the lossy mode resonance (LMR) phenomenon have attracted great interest from the scientific community due to their improved sensitivity and flexibility [33]. The deposition of a thin conducting metal oxide (CMO) layer with complex RI on optical waveguides produces various attenuation bands in the transmission spectra, and this process is known as the mode coupling of the optical waveguide modes and lossy modes of the thin absorbing layer at finite thickness [34]. The resultant mode coupling relies on the extensive overlapping between the mode fields and the phase-matching condition [35]. The phase matching state is met when the propagation constants (real part only) of both the waveguide mode and lossy mode become equal [36,37]. Therefore, when the lossy modes are close to the mode cut-off position, both conditions of LMR generation are met, and the lossy modes begin to guide through the CMO layer. The mode cut-off condition is additionally described by two key parameters, i.e., the incident light wavelength and thickness of the CMO layer [38]. Because of the lossy mode excitation, sharp minima in the transmitted LMR spectrum are produced due to the maximum transfer of energy from the optical waveguide mode to the lossy mode for a particular thickness of the CMO layer at discrete wavelengths. The equally significant technique used in sensing is surface plasmon resonance (SPR); though, there are few resemblances and differences between SPR and LMR. For SPR, the real part permittivity of the coated material should be negative and higher in magnitude than its own imaginary part. However, LMR is attained when the real part is positive and higher than the imaginary part. Indium tin oxide (ITO) is one of the CMOs that satisfies the generation condition of both SPR and LMR [39]. However, the resonance curves are wider in LMR-based FOSs when compared with SPR, leading to a decrease in the resolution of the measurements and hence, a decrease in the full width at half maximum and figure of merit. Importantly, the resonance dips in the SPR technique are mainly in the visible region of the electromagnetic spectrum; however, LMR dips may lie in both the visible and infrared regions.

A wide range of materials including CMOs, such as ITO [39], indium oxide (In_2_O_3_) [40], titanium dioxide (TiO_2_) [41], tin oxide (SnO_2_) [42], zinc oxide (ZnO) [43], etc., and polymers [44] support the LMR phenomenon, and these materials with large band gaps have shown exceptional structural, electronic, and mechanical properties [45,46]. Among them, ITO is the well-known CMO that has found many applications in optics and electronics [47]. It is formed by combining different composition ratios of In_2_O_3_ and SnO_2_, which impacts their electrical and mechanical properties. It has been reported in the literature that these ITO films are chemically stable and can be deposited on optical fibers using various deposition methods [44]. However, its conductivity can be further enhanced by increasing the thickness of the ITO layer, and therefore, designing a highly sensitive fiber optic LMR sensor with ITO is possible, which makes it a favorable contender to be used in the present study. Recently, ITO was also used for the fabrication of an electrochemical biosensor for the detection of antibiotic resistance genes in a water environment, and the results demonstrated a strong correlation with the electrochemical signals [48]. Another electrochemical sensor was proposed for the detection of sulfamethoxazole using a composite of graphene oxide/graphene over an ITO substrate [49]. We concluded that the ITO could lead to the enhancement of the electrochemical signals and the optical signals based on the LMR phenomenon. In the past, it has already been demonstrated that dual-domain sensing capability in electrochemical and optical domains can be offered by LMR sensors based on ITO film [50]. Moreover, ITO is assumed to be selective for the detection of the antibiotic ciprofloxacin. Indeed, ciprofloxacin and norfloxacin are the two kinds of fluoroquinolone antimicrobials [51], and recently, ITO was utilized for the selective detection of antibiotics norfloxacin [52]. In addition, to make the designed ITO-coated fiber optic LMR sensor more selective, a molecularly imprinted polymer (MIP) layer can be prepared over the ITO film without any further modifications [31].

Therefore, a novel design of an ITO-coated U-shaped fiber optic LMR biosensor is proposed for the detection of the antibiotic CIP. In the present study, ITO is utilized theoretically for the first time in the design of a U-shaped fiber optic LMR sensor. The U-shaped fiber structure is developed using the bending of an optical fiber, which further increases the penetration depth of the evanescent wave and hence, increases the sensitivity of the sensor when compared with a straight-core optical fiber probe of equal dimensions [53,54]. In the next section, a complete methodology for the proposed U-shaped fiber optic LMR sensor for RI sensing and antibiotic CIP hydrochloride is explained.

## 2. Theory

**Figure 2:**
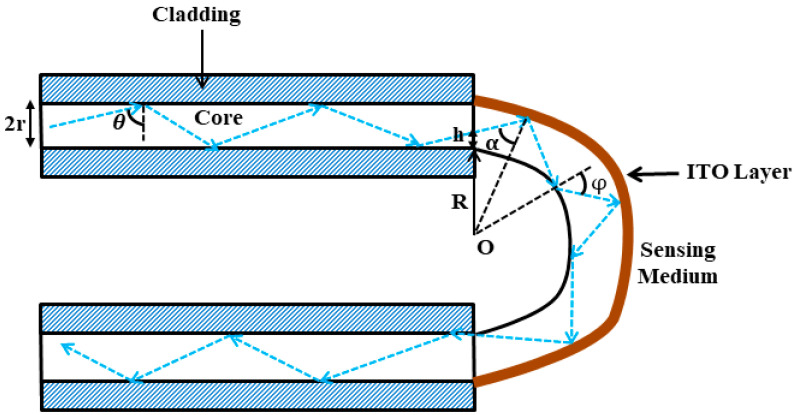
Graphical representation of the proposed ITO-coated U-shaped fiber optic LMR sensor.

In the present analysis, we have considered a step-index multimode optical fiber of core diameter (D) 600 µm and numerical aperture 0.24. The cladding is fully removed directly from the middle portion of the straight fiber using mechanical stripping tools such as a sharp tungsten blade. On heating the cladding-etched portion of the straight fiber, it is bent to form a ‘U’ shape which is also the length of the sensing region (L). For this, different conventional methods have been used in the past in which a heat source was used to soften the fiber to expedite the bending process and to create a perpetual bend in the form of a U-shape [55,56]. Therefore, the U-shaped fiber optic LMR sensing probe comprises an unclad inner bent region, i.e., the fiber core (Layer 1) with an optimum length of the lower region deposited with an ITO layer (Layer 2) and the sensing medium (Layer 3), as depicted in Figure 2. This LMR sensing probe is not drawn to scale because the fiber core radius (r) is much smaller than the bending radius (R) of the inner bent region and is designed to reflect the propagation of the rays within the core of the optical fiber.

### 2.1. Dispersion Properties

Layer 1 is assumed to be made of fused silica and hence, the wavelength dependency of its RI can be expressed as [57]:

(1) $$n_{core}\left(\lambda \right)=\sqrt{1+\frac{A_{1}\lambda^{2}}{\lambda^{2}-B_{1}^{2}}+\frac{A_{2}\lambda^{2}}{\lambda^{2}-B_{2}^{2}}+\frac{A_{3}\lambda^{2}}{\lambda^{2}-B_{3}^{2}}}$$

where λ is the wavelength of the incident light measured in units of µm and $A_{1}$, $A_{2}$, $A_{3},\;\;B_{1}$, $B_{2}$, and $B_{3}$ are the Sellmeier coefficients, and their mathematical values are given as: $\;A_{1}=0.6961663$,$\;A_{2}$ = 0.4079426,$\;A_{3}$ = 0.8974794, $B_{1}$ = 0.0684043, $B_{2}$ = 0.1162414, and $B_{3}$ = 9.896161.

The next layer (Layer 2) is composed of ITO, and its dispersion relation according to the Drude formula can be written as [58]:

(2) $$\;\;\;\;\varepsilon_{ITO}\left(\lambda \right)=\varepsilon_{real}+i\;\varepsilon_{imag.}=\varepsilon_{\infty }-\frac{\lambda^{2}\lambda_{c}}{\lambda_{p}^{2}\left(\lambda_{c}+i\;\lambda \right)}$$

where $\varepsilon_{\infty }=3.80$ and $\lambda_{c}$ and $\lambda_{p}$ represent the collision and plasma wavelengths, respectively, and their corresponding values are 11.21076 $\times 10^{-6}$ m and 5.6497 $\times 10^{-6}$ m. Finally, the third layer (Layer 3) consists of a sensing medium in the form of CIP hydrochloride solutions having different concentrations (0.001 to 0.029 mol∙dm^−3^) and different compositions (30%, 50%, and 70% in volume ratio *v*/*v*) in ethanol–water (E–W) mixtures at 30 °C. In that study, the authors used a thermostatically controlled Abbe Refractometer to measure the RI values of various concentrations of CIP hydrochloride as provided in Table 1 [59]. Furthermore, the concentration dependence of the refractive index (n) was studied using the following equation [60]:

(3) $$n=K\times c+n^{0}$$

where *K* is a constant measured in the units of dm^3^·mol^−1^ and depends on the chemical/physical properties of solute, *c* denotes the molar concentration of the antibiotic solution, and *n*^0^ is the RI at infinite dilution whose corresponding values for 30%, 50%, and 70% compositions are 1.3448, 1.3525, and 1.3552, respectively. Before calculating the numerical results for the CIP hydrochloride antibiotic, we have used the sensing medium refractive index (SMRI) values as 1.33 and 1.34 to optimize the ITO-film thickness (d), sensing region length (L), and bending radius (R) for an ITO-coated U-shaped fiber optic LMR sensor.

### 2.2. Estimation of Angles Inside the U-Shaped Region

To simplify the analysis and to obtain the maximum benefits of the U-shaped fiber geometry to enhance the evanescent field, a two-dimensional approach is considered in the present study. In this case, all the guided rays are assumed to be confined in the plane of bending. If *θ* measures the incident angle normal to the core–cladding interface in the straight region of the U-shaped LMR probe, then the subsequent transformed angles at the outer, α(h), and inner, φ(h), area of the U-shaped region can be estimated using the sine rule and are expressed as [61]:

(4) $$\alpha \left(h\right)={sin}^{-1}\left[\frac{\left(R+h\right)}{\left(R+2r\right)}sin\theta \right]$$

(5) $$\varphi \left(h\right)={sin}^{-1}\left[\frac{\left(R+h\right)}{R}sin\theta \right]$$

This phenomenon is known as the coupling of lower order modes to the higher order modes. Here, *h* measures the distance from the core–cladding interface of the inner U-bent region to the point where light ray reaches the entrance face of the outer U-bent region. Furthermore, if [$\theta_{1}$, $\theta_{2}$] is the range of incident angles of the rays launched at the input end of the U-shaped fiber, then the corresponding range of angles for the outer surface of the U-shaped region transforms to [$\alpha_{1}\left(h\right)$, $\alpha_{2}\left(h\right)$] inside the U-region and, thus, can be written as:

(6) $$\alpha_{1}\left(h\right)={sin}^{-1}\left[\frac{\left(R+h\right)}{\left(R+2r\right)}sin\theta_{1}\right]$$

(7) $$\alpha_{1}\left(h\right)={sin}^{-1}\left[\frac{\left(R+h\right)}{\left(R+2r\right)}sin\theta_{1}\right]$$

where$\;\theta_{1}={sin}^{-1}\left(\frac{n_{ITO}}{n_{core}}\right)$ and $\theta_{2}=\frac{\pi }{2}$, in which $n_{ITO}$ is the refractive index of the ITO layer which is calculated using the relation $n=\sqrt{\varepsilon }$. As the inner surface of the U-shaped region is not coated with the ITO layer, it is only used to transmit light using the phenomenon of total internal reflection (TIR).

### 2.3. Transmitted Power in the Case of a U-Shaped Fiber

In the proposed U-shaped fiber optic LMR sensor, light from a halogen light source is launched from one end of the U-shaped fiber using proper optics, and from another end, a detection system is attached to obtain the transmission spectra. Thus, the normalized transmitted power at the output end of the U-shaped fiber can be calculated as [62]:

(8) $$P_{trans\;}=\frac{\int_{0}^{2r}dh{\int_{\alpha_{1}(h)}^{\alpha_{2}(h)}R_{p}}^{N_{ref}(\theta )}\frac{n_{core\;}^{2}sin\theta cos\theta }{{\left(1-n_{core\;}^{2}{cos}^{2}\theta \right)}^{2}}d\theta }{\int_{0}^{2r}dh\int_{\alpha_{1}(h)}^{\alpha_{2}(h)}\frac{n_{core\;}^{2}sin\theta cos\theta }{{\left(1-n_{core\;}^{2}{cos}^{2}\theta \right)}^{2}}d\theta }\alpha$$

where $N_{ref}\left(\theta \right)$ denotes the total number of reflections made by the ray at the outer surface of the U-shaped fiber and can be written as:

(9) $$N_{ref}\left(\theta \right)=\frac{L}{8r}\left[cot\theta +cot\left(\left(\frac{R+2r}{R}\right)\right)\theta \right]$$

Moreover, $R_{p}{}^{N_{ref}\left(\theta \right)}$ in Equation (8) is the reflected power for the combination of both TM and TE polarized light, which can be calculated using the *N*-layer matrix model as given by [63,64]:

(10) $$R_{p}{}^{N_{ref}\left(\theta \right)}=\frac{{\left[R_{p}{}^{N_{ref}\left(\theta \right)}\right]}_{\mathrm{TM}}+{\left[R_{p}{}^{N_{ref}\left(\theta \right)}\right]}_{\mathrm{TE}}}{2}$$

Further, it should be noted that the light launched in an optical fiber sensor is unpolarized light. Thus, it is not possible to observe the separate effect of TM and TE polarized light in the designed fiber optic sensor experimentally. Therefore, the LMR curves obtained in the present analysis are a combination of both TM- and TE-polarized light. The number of layers (*N*) considered for the calculations in the present study is 3, i.e., Fiber core + ITO layer + sensing medium.

### 2.4. Sensing Parameters of the U-Shaped Fiber Optic LMR Sensor

Typically, the performance of a fiber optic LMR sensor is analyzed in terms of sensitivity, full width at half maximum (FWHM), the figure of merit (FOM), and the limit of detection (LOD). Initially, the sensitivity is described as the change in resonance wavelength ($\delta \lambda_{R}$) per unit change in SMRI, i.e., $\delta n_{s}$. It is computed in units of nm/RIU and defined by:

(11) $$\mathrm{Sensitivity}=\frac{\delta \lambda_{R}}{\delta n_{s}}$$

The next parameter is FWHM, which measures the central width of the transmitted LMR curve. It is analyzed in the units of nanometer and can be expressed by:

(12) $$\mathrm{FWHM}=\delta \lambda_{0.5}$$

Further, FOM is described as the ratio of sensitivity to the FWHM. Usually, it is evaluated in the units of RIU^−1^ and can be expressed as:

(13) $$\mathrm{FOM}=\frac{\mathrm{Sensitivity}}{\mathrm{FWHM}}$$

The last important parameter is LOD, which quantitatively determines the concentration of biomolecules in the sensing medium and numerically, it is defined by:

(14) $$\mathrm{LOD}=\frac{\Delta \lambda }{\mathrm{Sensitivity}}$$

where Δλ is the wavelength resolution of the spectrometer, which is considered as 1.10 nm in the present study. It is evaluated in the units of refractive index unit (RIU). In the upcoming section, a meticulous analysis of the numerical results attained for the proposed U-shaped fiber optic LMR sensor is presented.

## 3. Numerical Results and Discussion

As discussed earlier, we have considered a step-index multimode optical fiber for the theoretical analysis having a core diameter (D) of 600 µm and a numerical aperture of 0.24. In addition, ITO is considered an LMR active material to excite the lossy modes and is, therefore, systematically deposited on the outer surface of the U-shaped region. After this, the U-shaped LMR sensing probe coated with ITO film is exposed to a sensing environment. The whole simulation in this proposed study is accomplished using the newest version of MATLAB software. Next, we will find out the effect of changing the SMRI on the normalized transmitted LMR spectra.

### 3.1. Shift in LMR Curves

**Figure 3:**
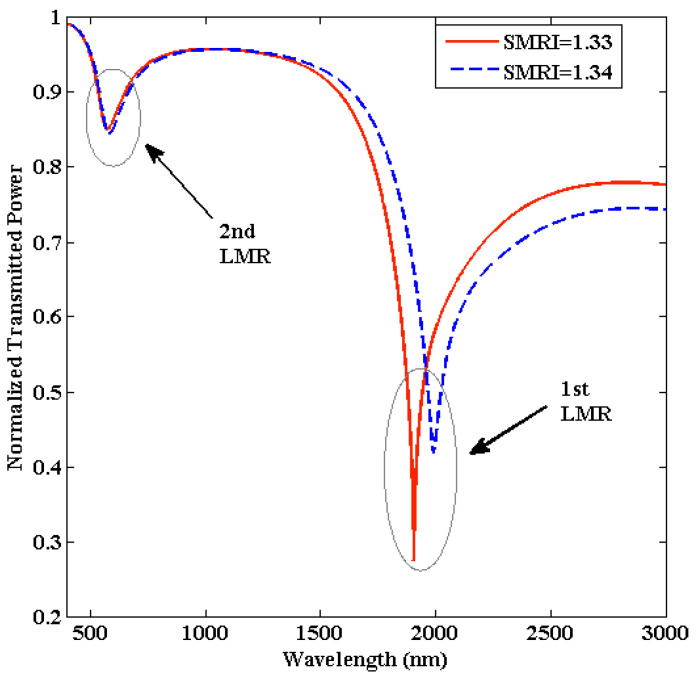
Transmitted LMR spectra for the proposed ITO-coated U-shaped fiber optic LMR sensor. Here, ITO thickness (d) = 90 nm, L = 0.5 cm, and R = 1.0 cm.

Figure 3 illustrates the transmitted LMR spectra for the proposed U-shaped fiber optic LMR sensor coated with an ITO layer. These LMR curves are achieved for two different SMRIs, i.e., 1.33 and 1.34, keeping the other parameters fixed (L = 0.5 cm and R = 1.0 cm). It is noted that for a certain value of SMRI, i.e., 1.33, the condition of LMR generation is fulfilled, and two LMRs can be seen in the spectrum at different resonance wavelengths.

In this condition, a complete transfer of energy from evanescent modes to lossy modes takes place, which creates multiple attenuation bands in the transmission spectra. The LMR curves attained at higher and lower wavelengths are identified as the first and second LMR, respectively, because these curves are due to the excitation of the first and second lossy mode, respectively, inside the ITO film. One can see that the first LMR lies in the near-infrared region of the electromagnetic spectrum, whereas the second LMR lies in the visible regime. By locating minima in the transmitted LMR spectra, the resonance wavelengths corresponding to the first and second LMR come out to be 1905.3 nm and 573.3 nm, respectively. If we increase the SMRI by an amount of 0.01 RIU, then a red shift in resonance wavelengths is achieved for both LMRs. For the first LMR, the resonance wavelength shifted to 1991 nm, which shows a red shift of 85.7 nm, whereas the equivalent shift for the second LMR comes out to be 7 nm. This shift in LMR wavelength occurs because if we enhance the SMRI, then the effective index of the multilayer structure is modified, and thus, the resonant excitation of the LMR takes place at some higher wavelength for both LMRs [65]. It is also remarked that the shift in the resonance wavelength is higher for the first LMR when compared with the second LMR, and hence, the sensitivity corresponding to the first LMR curve is much higher than the second LMR. It can also be understood in terms of the depth of resonance dip. From Figure 3, it is evident that if we move from the first LMR to the second LMR, a reduction in the depth of resonance dip is detected, which implies that the resonance strength is decreased, and hence, a smaller shift is observed in the case of the second LMR. The depth of a resonance dip is usually defined by the peak-to-valley value of the resonance dip [28]. We calculated that the depth of resonance for the first LMR is 0.683 when the SMRI is 1.33 and decreases to 0.542 when the SMRI was varied to 1.34. Similarly, the corresponding values of the second LMR were calculated to be 0.141 and 0.148 when the SMRI was equal to 1.33 and 1.34, respectively, which shows that the change in depth is insignificant.

**Figure 4:**
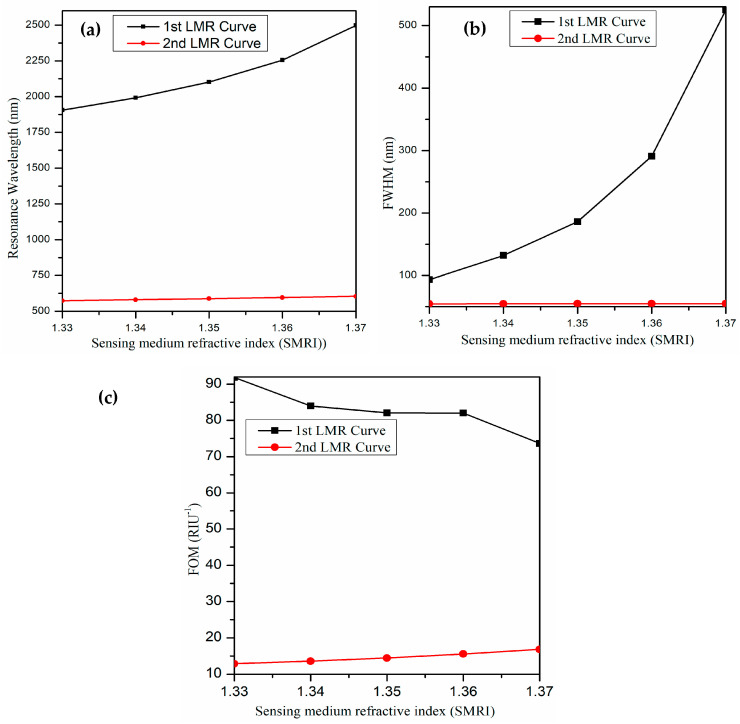
Variation in (**a**) resonance wavelength, (**b**) FWHM, and (**c**) FOM with sensing medium refractive index of both LMRs for the proposed U-shaped LMR sensor.

For the first LMR, the resultant sensitivity, FWHM, and FOM come out to be 8570 nm/RIU, 93.3 nm, and 91.85 RIU^−1^, respectively. Similarly, for the second LMR, the equivalent values are observed as 700 nm/RIU, 54.4 nm, and 12.86 RIU^−1^, respectively. As a result, we conclude that the overall performance of the first LMR is much higher than the second LMR, and hence, we will only concentrate on the results achieved for the first LMR in the imminent sections. It is also observed that the proposed U-shaped LMR sensor works for a wide range of SMRIs from 1.33 to 1.37. Figure 4a portrays the behavior of resonance wavelengths with SMRI for each LMR curve. It is evident that for the first LMR, the variation in resonance wavelength is linear in the given range of SMRIs, and it increases with an increase in SMRI values. On the other hand, the corresponding variation in resonance wavelength is very small for the second LMR curve. In addition, the resonance wavelength for the first LMR lies at higher wavelength values than the second LMR. Similarly, the variations in FWHM and FOM with SMRI for each LMRs have been depicted in Figure 4b,c. In the next section, we will optimize the thickness of the ITO film to attain a better performance of the U-shaped fiber optic LMR sensing probe.

### 3.2. Optimization of the ITO film Thickness (d)

**Figure 5:**
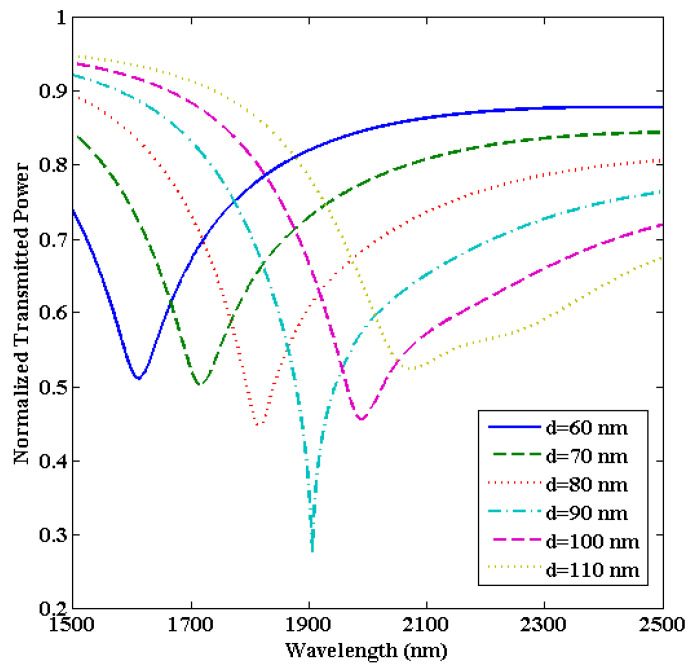
Transmitted LMR spectra (only the first LMR curve) for various thicknesses of the ITO film coated over the U-shaped fiber optic sensing probe. Here, SMRI = 1.33, L = 0.5 cm, and R = 1.0 cm.

Here, we will vary the thickness of the ITO film while keeping the other parameters fixed, i.e., L = 0.5 cm and R = 1.0 cm. For an ITO layer having a thickness below 60 nm, we observe that only one LMR curve is achieved in the transmission spectra. However, from the description of lossy modes, it is evident that the number of excited lossy modes depends on the thickness of the coated metal oxide layer [66], which implies that multiple LMRs are generated in the transmission spectrum if we increase the thickness of the ITO film. Earlier, we established that the first LMR is much more sensitive than the second LMR, so we will focus on the first LMR only. Figure 5 depicts the normalized transmitted LMR spectra (only the first LMR curve) for various thicknesses of the ITO film coated over the U-shaped fiber having an SMRI of 1.33.

It is found that for ITO-film having a thickness of 60 nm, the second LMR lies at a resonance wavelength of 1611 nm. If we further increase the thickness of the ITO film up to 90 nm, deeper LMR curves (i.e., higher depth of resonance dip) with a red shift in resonance wavelengths are attained. For an ITO thickness larger than 90 nm, wide and distorted LMR spectra are achieved, which makes it practically impossible to detect the resonance wavelengths, and hence, the calculation of sensitivities. It is measured that for an ITO thickness of 90 nm, the resonance wavelength lies at 1905 nm, and this LMR curve is much better than the LMR curves observed at other ITO thicknesses. The performance parameters for the first LMR curve were evaluated for various ITO thicknesses, which are shown in Table 2.

**Figure 6:**
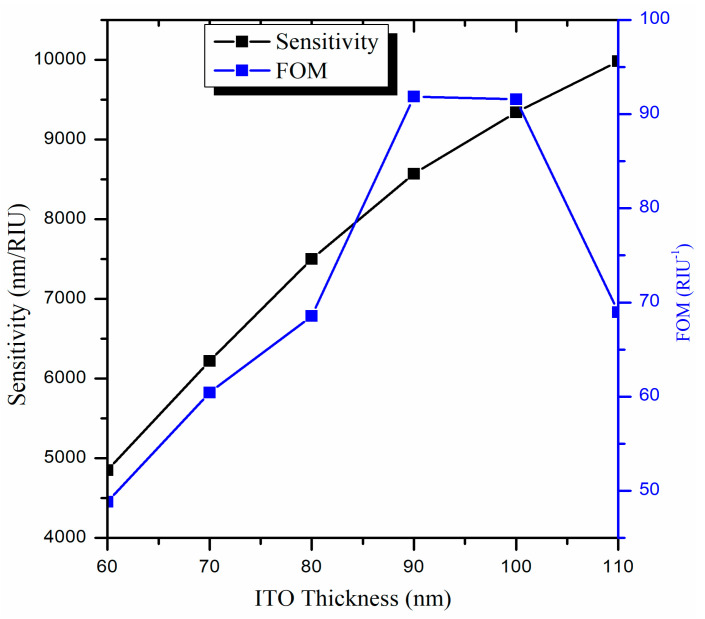
Variation in the sensitivity and figure of merit for various thicknesses of the ITO layer coated over the U-shaped LMR sensing probe.

The calculated results show that for an ITO thickness of 60 nm, the corresponding value of sensitivity, FWHM, and FOM come out to be 4850 nm/RIU, 99.3 nm, and 48.84 RIU^−1^, respectively. To design a better LMR sensor, the sensitivity and FOM should be high, and the FWHM should be low. This is because the higher the FWHM, the wider will be the LMR curve and hence, the lower will be the FOM values. The purpose of the designed U-shaped LMR sensor is to make it as efficient as possible. To understand this, the variation in sensitivity and FOM values for various ITO thicknesses have been plotted in Figure 6. One can see that the FOM increases with the increase in thickness, attains a maximum value at 90 nm, and then decreases rapidly. As a result, the thickness of the ITO film is optimized to 90 nm for the proposed U-shaped fiber optic LMR sensor. For this thickness, the sensitivity, FWHM, and FOM are detected to be 8570 nm/RIU, 93.3 nm, and 91.85 RIU^−1^, respectively. In the next section, the sensing region length (L) will be optimized for the designed U-shaped fiber optic LMR sensor.

### 3.3. Optimization of the Sensing Region Length (L)

**Figure 7:**
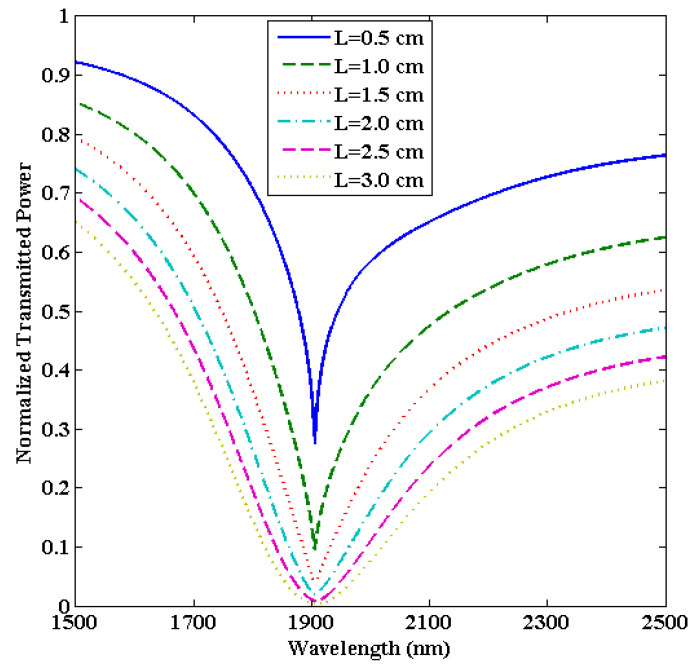
Transmitted LMR spectra (only the first LMR curve) for various sensing region lengths (L) of an ITO-coated U-shaped fiber optic LMR sensor. Here, SMRI = 1.33, d = 90 nm, and R = 1.0 cm.

In this section, we will alter the sensing region length (L) while keeping the other parameters fixed (i.e., d = 90 nm and R = 1.0 cm). Figure 7 depicts the transmitted LMR spectra (only the first LMR curve) for various sensing region lengths in an ITO-coated U-shaped optical fiber having an SMRI of 1.33.

**Figure 8:**
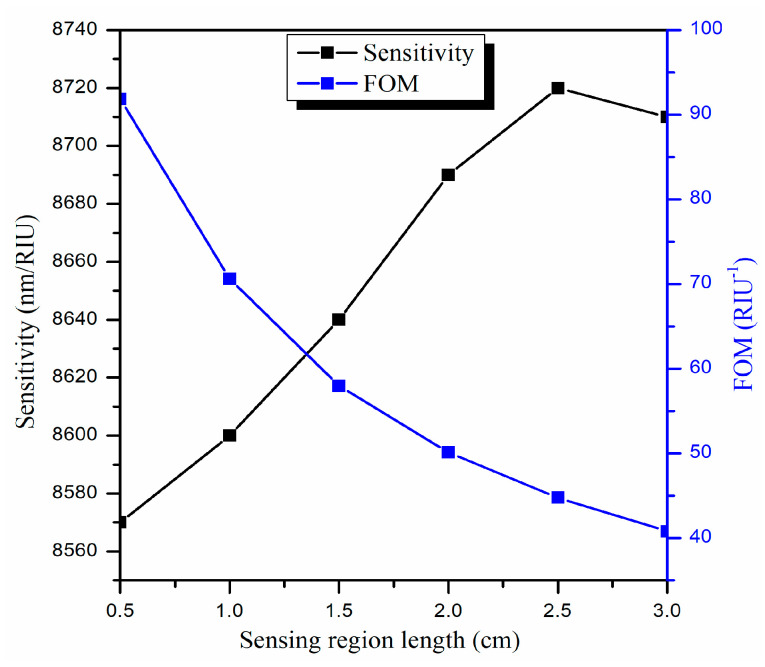
Variation in the sensitivity and figure of merit with various sensing region lengths (L) for the designed U-shaped LMR sensing probe.

For L = 0.5 cm, a sharp LMR curve is attained at resonance wavelength 1906 nm, and the corresponding sensitivity is calculated to be 8570 nm/RIU. It is noticed that the LMR curve becomes broader if we further increase L, but there is no change in the resonance wavelengths. This implies that the central width of the LMR curve increases for higher L, which in turn increases the FWHM and hence decreases the FOM values. This is because a higher L leads to more loss in the propagating light inside the U-shaped fiber which needs a high-power light source to balance, and the fiber turns out to be more fragile [67]. For example, if L = 3.0 cm, then the corresponding sensitivity, FWHM, and FOM come out to be 8710 nm/RIU, 213.5 nm, and 40.79 RIU^−1^, respectively. This means that if we increase L = 0.5 cm to 3.0 cm, then the sensitivity increases from 8570 nm/RIU to 8710 nm/RIU, which is a very little improvement (1.6%), while the FOM shows a strong decrement from 91.85 RIU^−1^ to 40.79 RIU^−1^ (56%) as depicted in Figure 8. Hence, the overall performance of the designed U-shaped fiber optic LMR sensor decreases for higher L values. For this reason, we have chosen the optimized value of L as 0.5 cm because, for higher L values, the sensitivity increases to a small extent, but the other parameters decrease sharply and, as we already discussed, the primary objective of the proposed U-shaped LMR sensor is to make it as compact as possible. In the upcoming section, the bending radius (R) will be optimized for the designed U-shaped fiber optic LMR sensor coated with an ITO layer.

### 3.4. Optimization of the Bending Radius (R)

Here, we will change the bending radius (R) while keeping the other parameters fixed (i.e., d = 90 nm and L = 0.5 cm). Since the bending radius of the U-shaped fiber is classified into inner and outer bending radii, here we will only focus on the inner bending radius to optimize the performance of the designed U-shaped LMR sensor.

**Figure 9:**
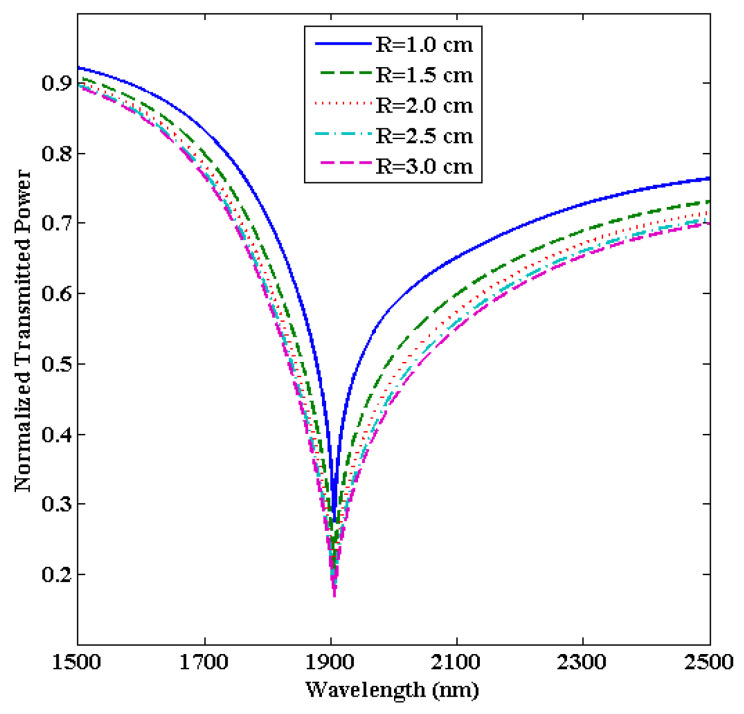
Transmitted LMR spectra (only the first LMR curve) for various bending radii of an ITO-coated U-shaped fiber optic LMR sensor. Here, SMRI = 1.33, d = 90 nm, and L = 0.5 cm.

**Figure 10:**
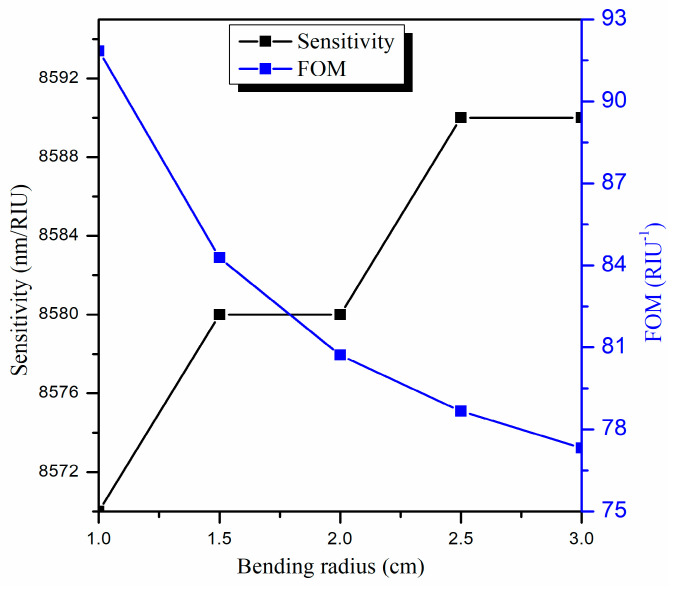
Variation in the sensitivity and figure of merit with various bending radii (R) for the designed U-shaped LMR sensing probe.

Next, we compare the results obtained for the proposed U-shaped LMR sensor to the straight-core LMR sensor both coated with a thin ITO film. We observed that the maximum sensitivity in the present case comes out to be 8570 nm/RIU, which is much higher than the sensitivity (1221 nm/RIU) attained for the straight-core optical fiber LMR sensor [68]. This is because in the case of a U-shaped fiber, the evanescent wave has a larger value of penetration depth, and hence, there is a stronger coupling of the evanescent modes with the lossy modes, which in turn provides a higher sensitivity value. Moreover, for the straight-core fiber optic probe, the angle of ray propagation is constant, and hence, the number of reflections and absorption coefficient depends only on the radius of the fiber core. On the other hand, in the case of a U-shaped fiber structure, the angle of ray propagation is not fixed and instead decreases. Figure 9 displays the normalized transmitted LMR spectra (only the first LMR curve) for various bending radii in a U-shaped fiber with an SMRI of 1.33. It is observed that if we increase the bending radius from R = 1.0 to 3.0 cm, then the sensitivity increases from 8570 nm/RIU to 8590 nm/RIU, which is insignificant, but the LMR curve becomes broader. This means the central width of the LMR curve, i.e., FWHM, increases, which in turn decreases the FOM values. On changing the bending radius from 1.0 to 3.0 cm, the FWHM increases from 93.3 nm to 111.1 nm, and the FOM decreases from 91.84 RIU^−1^ to 77.31 RIU^−1^, as shown in Figure 10. Therefore, we have chosen the optimized value of the bending radius (R) as 1.0 cm because, for a higher bending radius, there is no increment in sensitivity, but the sensor accuracy and other parameters become low. Thus, for the proposed U-shaped fiber optic LMR sensor, the parameters are optimized to d = 90 nm, L = 0.5 cm, and R = 1.0 cm. In the next section, the proposed ITO-coated U-shaped LMR sensing probe will be used for the detection of the antibiotic CIP hydrochloride in ethanol–water mixtures.

### 3.5. Detection of the Antibiotic Ciprofloxacin (CIP)

**Figure 11:**
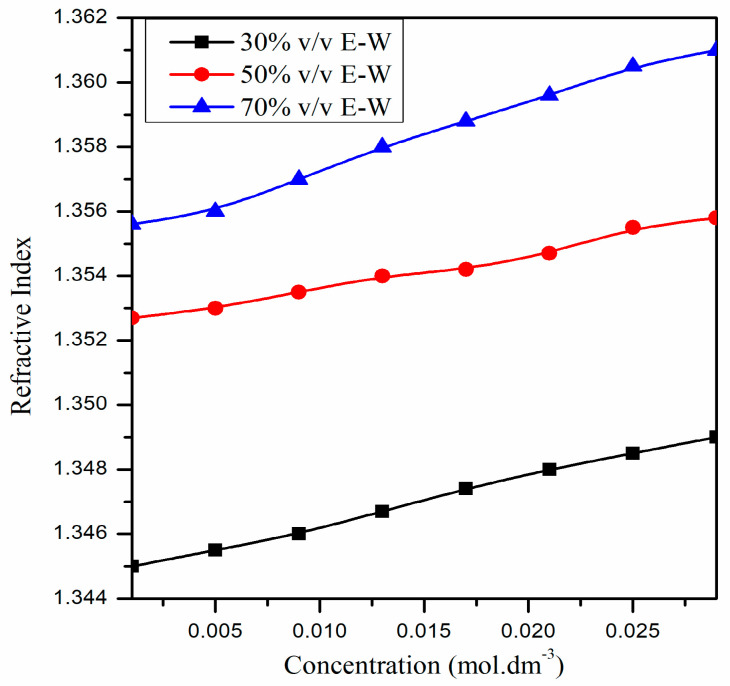
Variation in the RI with the concentration of CIP hydrochloride solutions for different E–W mixtures [59].

**Figure 12:**
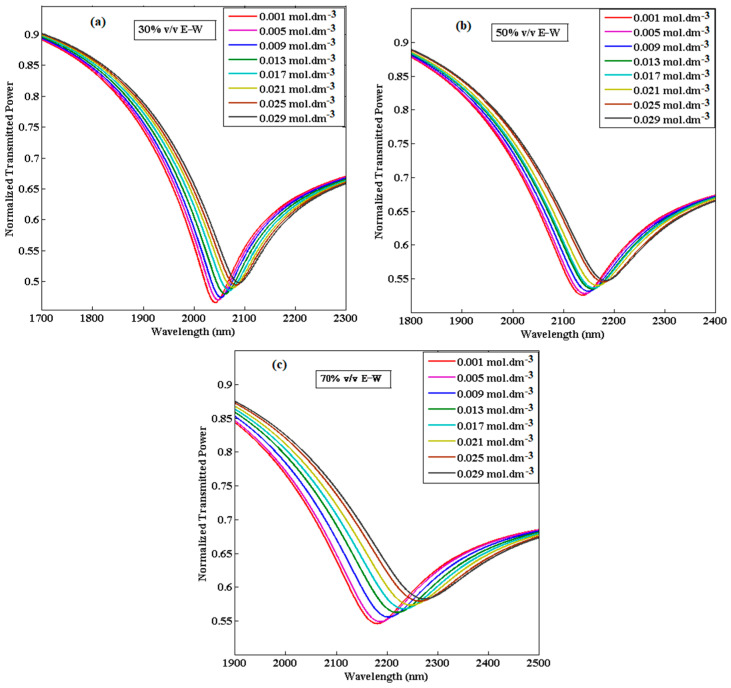
Transmitted LMR spectra (the first LMR curve) of the proposed ITO-coated U-shaped LMR sensor for the detection of ciprofloxacin having different concentrations and compositions (**a**) 30% *v*/*v* E–W mixture, (**b**) 50% *v*/*v* E–W mixture, and (**c**) 70% *v*/*v* E–W mixture.

Until now, we have only provided the RI sensing application of the proposed U-shaped fiber optic LMR sensor. The variation in the RI with the concentration of CIP hydrochloride solutions is linear for each E–W mixture, as depicted in Figure 9, and their RI values lie between 1.345 and 1.361. Therefore, the proposed ITO-coated U-shaped fiber LMR biosensor can be utilized to detect a low concentration of CIP hydrochloride (0.001 to 0.029 mol∙dm^−3^) in E–W mixtures. From Figure 11, it is remarked that with an increase in the concentration of CIP hydrochloride, RI also increases for each E–W mixture. For example, when the concentration of CIP hydrochloride is 0.001 mol∙dm^−3^ for a 30% *v*/*v* E–W mixture, its RI is 1.345, but it changed to 1.349 when the concentration was increased to 0.029 mol∙dm^−3^. This difference in RI values may be due to the change in structure/composition of CIP with the addition of ethanol–water. Furthermore, the RI increases with the increase in vol% of ethanol in solution for a given CIP hydrochloride concentration. Figure 12a–c displays the transmitted LMR spectra (the first LMR curve only) of the proposed ITO-coated U-shaped LMR sensor when the different concentration of CIP hydrochloride solutions is placed around that sensing layer. The results demonstrate that with the increase in the concentration of CIP hydrochloride, a red shift in resonance wavelength is seen for each E–W mixture, and these LMR curves become broader with an increase in the concentration of CIP. This red shift in resonance wavelength is due to an increase in the RI values with the concentration of CIP hydrochloride, and hence, the condition of LMR generation is satisfied at some higher wavelength values.

**Figure 13:**
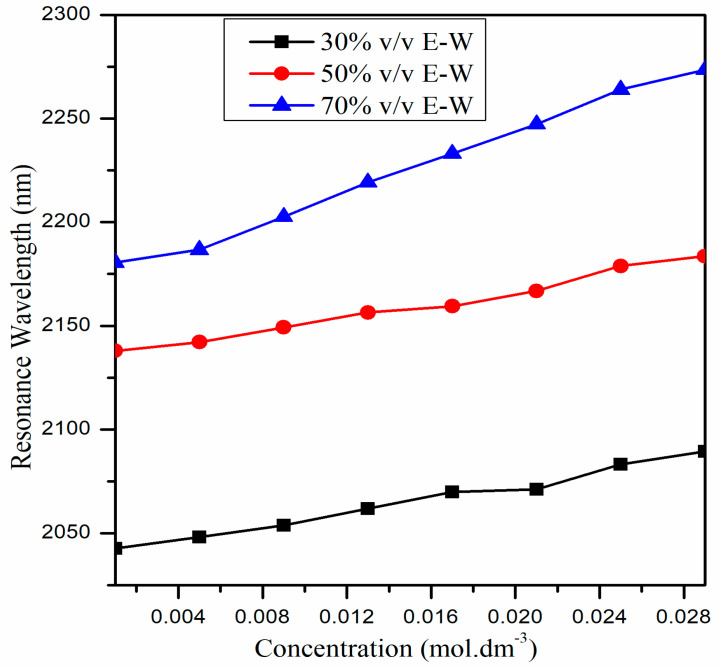
Variation in the resonance wavelength with the concentration of CIP hydrochloride for the proposed ITO-coated U-shaped LMR sensor.

On comparing the results attained for various E–W mixtures, it is observed that for the 30% *v*/*v* E–W mixture, the resonance wavelengths lie at lower wavelength values and move to higher wavelength values (i.e., 50% *v*/*v* and 70% *v*/*v*) if we increase the vol% of ethanol in solution for a particular concentration of CIP hydrochloride. For the designed sensor, the calibration curves showing the variation in resonance wavelength with the increased concentration of CIP hydrochloride are depicted in Figure 13.

It is evident that resonance wavelength shows linear increment with the increase in concentration for each mixture. However, the resonance wavelengths are higher for the 70% *v*/*v* E–W mixture when compared with the rest of the two mixtures. In addition, the shift in resonance wavelength is higher for the 70% *v*/*v* E–W mixture, followed by the 50% and 30% *v*/*v* E–W mixtures. Therefore, the sensitivity is higher for the 70% *v*/*v* E–W mixture and is minimum for the 30% *v*/*v* E–W mixture. On further evaluating the performance of the designed U-shaped LMR biosensor, we observed that the resultant value of sensitivity, FWHM, FOM, and LOD for the 30% *v*/*v* mixture are 11,270.4 nm/RIU, 127.7 nm, 91.42 RIU^−1^, and 9.76 × 10^−5^ RIU, respectively. In an identical manner, for the 50% *v*/*v* E–W mixture, the subsequent values are found to be 14,744.4 nm/RIU, 160.4 nm, 91.90 RIU^−1^, and 7.46 × 10^−5^ RIU, respectively, and for 70%, these values are 17,209.9 nm/RIU, 193.4 nm, 89.14 RIU^−1^, and 6.39 × 10^−5^ RIU, respectively. Therefore, the highest sensitivity for the proposed ITO-coated U-shaped LMR biosensor for the detection of the antibiotic CIP hydrochloride is achieved at 17,209.9 nm/RIU, the highest FOM at 91.42 RIU^−1^, and the LOD at 6.3 × 10^−5^ RIU. In addition, a sensitivity comparison of the proposed U-shaped LMR sensor with the recently published fiber optic LMR sensors is shown in Table 3.

## 4. Conclusions

In this paper, we proposed an ITO-coated U-shaped fiber optic LMR biosensor for their usage in RI sensing applications and for antibiotic CIP detection. The design and evaluation of the LMR sensor were performed using a transfer matrix method-based simulation platform, and the effect of ITO thickness was observed on the performance of the designed U-shaped LMR sensor. It was concluded that the 90 nm thick ITO layer was able to generate two better LMRs in the transmission spectrum than the other ITO thicknesses, and the other parameters such as sensing length and bending radius were optimized to 0.5 cm and 1.0 cm, respectively. Moreover, it was also proven that the first LMR curve is always more highly sensitive than the rest of the LMR curves achieved in the normalized transmitted LMR spectra. The theoretical results revealed that the designed U-shaped LMR biosensor possesses a maximum sensitivity of 17,209.9 nm/RIU with the highest FOM of 91.42 RIU^−1^ and LOD of 6.3 × 10^−5^ RIU for the detection of CIP hydrochloride in the concentration range of 0.001 to 0.029 mol∙dm^−3^. Hence, the sensitivity of the proposed U-shaped LMR sensor with optimized parameters was observed to be around seven times higher than that of the conventional fiber optic LMR sensor.

## Figures and Tables

**Table 1:** RI Values of CIP hydrochloride solutions for various concentrations and compositions [59].

Concentration of CIP Hydrochloride (mol∙dm^−3^)	Refractive Index (n)		
	30% *v*/*v* E–W	50% *v/v* E–W	70% *v*/*v* E–W
0.001	1.3450	1.3527	1.3556
0.005	1.3455	1.3530	1.3560
0.009	1.3460	1.3535	1.3570
0.013	1.3467	1.3540	1.3580
0.017	1.3474	1.3542	1.3588
0.021	1.3480	1.3547	1.3596
0.025	1.3485	1.3555	1.3605
0.029	1.3490	1.3558	1.3610

**Table 2:** Calculation of performance parameters corresponding to the first LMR curve for various thicknesses of the ITO film coated over the U-shaped optical fiber.

ITO Thickness (nm)	Performance Parameters		
	Sensitivity (nm/RIU)	FWHM (nm)	FOM (RIU^−1^)
60	4850	99.3	48.84
70	6220	102.9	60.44
80	7500	109.4	68.55
90	8570	93.3	91.85
100	9340	102.0	91.56
110	9980	144.7	68.97

**Table 3:** Sensitivity comparison of the designed LMR sensor with previously reported LMR/SPR sensors.

Type of LMR/SPR Configuration	Theoretical/Experimental	Maximum Sensitivity (nm/RIU)	Figure of Merit (RIU^−1^)	References
Uniform fiber core + ITO	Experimental	1221	-	Lopez et al. [68]
Uniform fiber core + In_2_O_3_	Experimental	4068	-	Zamarreno et al. [69]
Uniform fiber core + AZO	Experimental	2280	-	Ozcariz et al. [70]
Tapered fiber core + AZO/TiO_2_	Theoretical	9000	-	Paliwal et al. [71]
D-shaped fiber + TiO_2_	Experimental	4122	-	Tien et al. [72]
D-shaped fiber + IGZO	Experimental	12,929	396.20	Ozcariz et al. [73]
Uniform fiber core + CuO	Experimental	7324	-	Ozcariz et al. [74]
U-shaped fiber core + gold + graphene	Theoretical	15,000	33.59	Xie et al. [75]
U-shaped fiber core + graphene + silver NPs	Experimental	1198	-	Zhang et al. [76]
U-shaped fiber core + ITO	Theoretical	17,209	91.42	Present work

## Data Availability

All the data generated/analyzed in this study is included in this article itself.
